# Change Process in Psychotherapy for Depressed Inpatient: A Case Within Trial Study

**DOI:** 10.1192/j.eurpsy.2023.807

**Published:** 2023-07-19

**Authors:** K. Tzartzas, Y. de Roten, G. Ambresin

**Affiliations:** 1 Département des Policliniques (DDP), Unisanté - Centre de médecine générale et de santé publique; 2 University Institute of Psychotherapy, Department of Psychiatry, Lausanne University Hospital, Lausanne, Switzerland

## Abstract

**Introduction:**

Results of a randomized controlled trial supported the efficacy of a manualized, Intensive and Brief Psychodynamic Psychotherapy (IBPP) for inpatients with severe depression, but the mechanisms by which the interaction between a psychotherapist and a patient can be involved in a process of change require more direct study.

**Objectives:**

The study aimed to explore how the psychotherapist and the patient interacted to work through the themes of focalization of their therapeutic work and how their work was part of a potential process of change.

**Methods:**

A pragmatic case study was conducted on two cases selected from the umbrella study with one responder and one nonresponder to treatment (response defined as > 46% decrease in depressive symptoms on the MADRS). For each case, the verbatims of 6 sessions were analyzed, focusing on the themes of the IBPP manual.

**Results:**

Two main functions were revealed: 1) “**Becoming the subject of one’s depression”**, which includes the following themes: i) “Following the Tracks of Pain and Loss”; ii) “Negotiating the Distance to the Cemetery”; iii) “Beginning to Accept”; iv) “Investing in New Projects “; and 2) **“Regaining a sense of support”** which includes the following themes: i) “Not Being Beaten Down”; ii) “Emptying a Full Closet”; iii) “Fear of Ending Up Alone”. The supportive interactions (regaining a sense of support) were present in a similar way in both cases, whereas the specific interactions (becoming the subject of one’s depression) were more present in the responder case.

**Conclusions:**

In the psychotherapy of inpatients with severe depression, specific therapeutic interventions aiming to mobilize internal processes of symbolization, comprehension, and appropriation are necessary to reactivate a previously frozen mourning process. However, such interventions should be carried out in conjunction with interactions aiming to help the patient regain a sense of support. The central role of interactions that serve to build a therapeutic space and to restore epistemic trust was an unexpected result. It invites psychotherapists to pay particular attention to acknowledging a patient’s melancholic suffering, and to continuously seek to adjust their interventions to foster the continuity of emotional contact and the emergence of a sense of support. Theoretical and clinical implications of these findings will be discussed.

**Disclosure of Interest:**

None Declared

